# Nanoparticles Loaded with Docetaxel and Resveratrol as an Advanced Tool for Cancer Therapy

**DOI:** 10.3390/biomedicines10051187

**Published:** 2022-05-20

**Authors:** Magdalena Jurczyk, Janusz Kasperczyk, Dorota Wrześniok, Artur Beberok, Katarzyna Jelonek

**Affiliations:** 1 Centre of Polymer and Carbon Materials, Polish Academy of Sciences, Curie-Skłodowska 34 St., 41-819 Zabrze, Poland; d200967@365.sum.edu.pl (M.J.); janusz.kasperczyk@sum.edu.pl (J.K.); 2 Department of Pharmaceutical Chemistry, Faculty of Pharmaceutical Sciences in Sosnowiec, Medical University of Silesia, Jagiellońska 4, 41-200 Sosnowiec, Poland; dwrzesniok@sum.edu.pl (D.W.); abeberok@sum.edu.pl (A.B.); 3 Department of Biopharmacy, Faculty of Pharmaceutical Sciences in Sosnowiec, Medical University of Silesia, Jedności 8, 41-200 Sosnowiec, Poland

**Keywords:** docetaxel, resveratrol, nanoparticles, co-delivery, nanoparticles, anticancer drugs, chemosensitizer, drug delivery, targeted delivery

## Abstract

A growing interest in the use of a combination of chemosensitizers and cytostatics for overcoming cancer resistance to treatment and the development of their delivery systems has been observed. Resveratrol (Res) presents antioxidant, anti-inflammatory and chemopreventive properties but also limits multidrug resistance against docetaxel (Dtx), which is one of the main causes of failure in cancer therapy with this drug. However, the use of both drugs presents challenges, including poor bioavailability, the unfavourable pharmacokinetics and chemical instability of Res and the poor water solubility and dose-limiting toxicity of Dtx. In order to overcome these difficulties, attempts have been made to create different forms of delivery for both agents. This review is focused on the latest developments in nanoparticles for the delivery of Dtx, Res and for the combined delivery of those two drugs. The aim of this review was also to summarize the synergistic mechanism of action of Dtx and Res on cancer cells. According to recent reports, Dtx and Res loaded in a nano-delivery system exhibit better efficiency in cancer treatment compared to free drugs. Also, the co-delivery of Dtx and Res in one actively targeted delivery system providing the simultaneous release of both drugs in cancer cells has a chance to fulfil the requirements of effective anticancer therapy and reduce limitations in therapy caused by multidrug resistance (MDR).

## 1. Introduction

According to the World Cancer Report, cancer is the leading cause of premature death in 134 out of 183 countries (in people aged 30–69) and ranks third or fourth in the remaining 45 countries [1]. The clinical efficacy of current treatment methods, such as oncological surgery, chemotherapy and radiotherapy, remains limited, and in the case of tumours that cannot be removed surgically, chemotherapy is often the only treatment option. A major problem with intravenous systemic chemotherapy is non-specific tumour targeting and the difficulty in achieving therapeutic drug levels within the tumour or in the vicinity of the tumour [2]. In the case of paclitaxel (Ptx) administered by intravenous infusion, less than 0.5% of the total dose is locally available within the tumour. In addition, high drug concentrations are observed in healthy tissues, leading to severe side effects and dose-limiting toxicity [3].

**Figure 1:**
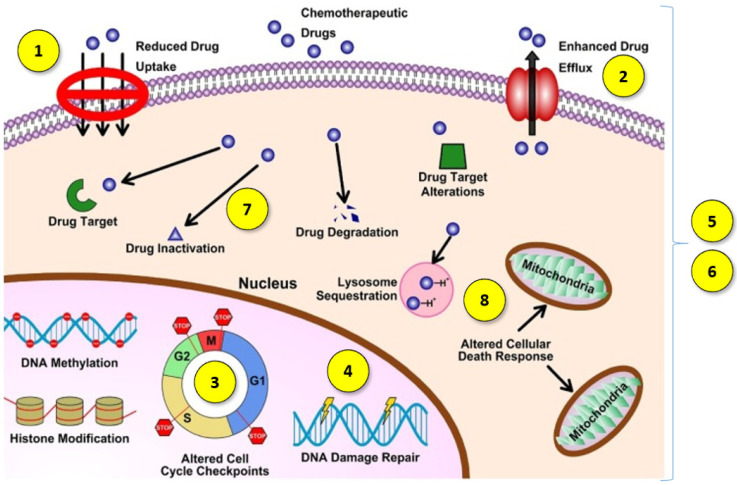
Diagram of multidrug resistance mechanisms. Reprinted and adapted with permission from Ref. [5] Copyright (2021) Elsevier.

Another challenge in cancer treatment is overcoming multidrug resistance (MDR). MDR is the complex phenomenon of the chemoresistance of neoplastic cells to treatment, which can occur not only as a result of cytostatic drug treatment but can also be a feature of cancer untreated with chemotherapy. Moreover, chemoresistance after the administration of one drug may cause resistance to many other anticancer agents via different structures and mechanisms of action [4]. This is a broad mechanism, which may involve the development of one or more features by cancer cells (Figure 1):

(1) Decreased drug absorption by cancer cells [5];
(2) The increased expression of certain ATP-binding cassette efflux transporters, including P-glycoprotein (P-gp/ABCB1), multidrug resistance protein 1 (MRP1/ABCC1) and BCRP (ABCG2), which lower the cytosolic concentration of the active agents through increased drug transportation outside the cell [4];
(3) The impaired function of pro-apoptotic factors, resulting in cancer cells avoiding programmed death [6];
(4) A better ability to repair damaged DNA [6];
(5) Qualitative or quantitative changes in specific cell targets [5];
(6) Changes that allow cancer cells to tolerate adverse or stressful conditions caused by treatment with antineoplastic agents by transforming them into less effective or inactive metabolites [7];
(7) An increasing in the efficiency of the metabolism and biotransformation of cytostatic drugs, leading to their conversion into metabolites without cytostatic effect [8];
(8) The intracellular and intercellular sequestration of drugs in well-defined organelles away from the cellular target, including the lysosomal compartmentalization of hydrophobic, weakly basic anticancer drugs [9].

The most commonly used cytostatic drugs, including methotrexate, Ptx, docetaxel (Dtx), vincristine, etoposide, 5-fluorouracil, doxorubicin, daunorubicin, cytarabine and many others, have been found to be resistant to treatment [10]. Therefore, an important goal is to create a therapeutic agent, drug or drug formulation that can overcome these problems and ensure greater clinical effectiveness in anticancer therapy [11]. Currently, effective delivery systems for traditional chemotherapeutic agents as well as combinations of these drugs with chemosensitizing agents (also of natural origin) have been developed. Additionally, the use of naturally occurring plant molecules appears to be a cost-effective way to overcome the resistance of cancer to traditional treatment. However, a considerable challenge in the use of chemosensitizers is the genetic variation between populations, e.g., in gynecological cancer, the multigenic basis of tumours and the accompanying genetic polymorphism that affect the effectiveness of chemosensizitaion and constitutes a significant challenge in the development of an effective treatment [12].

One of the combinations with beneficial therapeutic effects is the co-administration of resveratrol (Res) with docetaxel (Dtx). According to recent outcomes, in addition to the antioxidant, antiinflammatory and chemopreventive properties of Res [13], it also limits multidrug resistance against Dtx [14], which is one of the main causes of failure in cancer therapy with this drug. However, the use of both drugs presents the challenges of poor bioavailability, the unfavourable pharmacokinetics and chemical instability of Res and the poor water solubility and dose limiting toxicity of Dtx. In order to overcome these difficulties, attempts have been made to create different forms of delivery for both agents, which are described below. This review is focused on the latest developments, i.e., published in the last 10 years, in nanoparticles for the delivery of Dtx, Res and for the combined delivery of these two drugs. The aim of this review was also to summarize the synergistic mechanism of action of Dtx and Res on cancer cells. This subject is important and current considering the increasing interest in the use of a combination of chemosensitizers and cytostatics for overcoming cancer resistance to treatment and the development of their delivery systems.

## 2. Nanoparticles for Drug Delivery Application

The use of nanoparticles (NPs) as drug delivery systems has received much attention in the context of cancer treatment in recent years. NPs allow for the improvement of the pharmacokinetics of poorly soluble hydrophobic drugs and cancer-specific drug delivery by passive or active targeting strategies, which can promote their preferential accumulation in tumour tissues [15]. Nanocarriers are characterized by their nanometric size (<1000 nm), high surface area-to-volume ratio and favourable drug release profiles [16]. Therapeutic agents can either be encapsulated into nanocarriers or covalently bonded or adsorbed onto the surfaces of these carriers [17]. The advantages of NPs include enhancing the safety, pharmacokinetic profiles and bioavailability of the administered drugs, leading to improved therapeutic efficacy compared to conventional therapy. Nanoparticles can be synthesized from various organic or inorganic materials such as lipids, proteins, synthetic/natural polymers and metals. NPs can be classified into several groups, such as polymeric nanoparticles, liposomes, dendrimers, micelles and inorganic NPs, based on the components used for synthesis or the structural aspects of the NPs [17,18,19].

**Figure 2:**
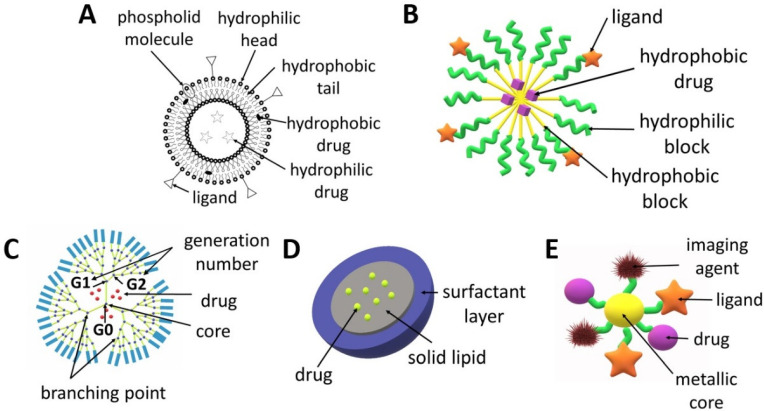
Scheme of the structures of drug delivery systems: (**A**) liposome, (**B**) micelle, (**C**) dendrimer, (**D**) solid lipid nanoparticle, (**E**) metallic nanoparticle.

Liposomes (Figure 2A) are spherical structures made of phospholipids and cholesterol that form a bilayer, which enclose a central space for the encapsulation of various molecules ranging from small to large in terms of molecular weight such as DNA or drugs. The main advantages of liposomes include a high drug loading capacity, the possibility of the simultaneous delivery of hydrophilic and hydrophobic active agents and the fact that the entrapment of molecules in liposomes increases their circulation time in the blood [20]. The structure of liposomes can be easily modified in order to obtain the expected therapeutic effect [21]. In addition to an active targeting strategy which uses ligands (both small molecule ligands and antibodies), liposomes can be designed in such a way so that the drug is released in a specific tumour microenvironment or as a result of strictly defined conditions, e.g., under the influence of light, enzymes, pH or ultrasound [22]. The possibility of using various strategies to target liposomes at cancer cells reduces the risk of the negative side effects of chemotherapy on healthy cells. The choice of the preparation method for liposomes is determined by the desired properties of the liposomes (e.g., size, half-life), repeatability of the process, cost, properties of the liposome components used for synthesis, properties of the encapsulated drugs, type of solvent and liposome application processes. The most frequently used technique for liposome production is thin-film hydration, which involves dissolving lipids in an organic solvent followed by its evaporation and then the dispersing of the obtained film in water. The disadvantages of this method are the low efficiency in terms of drug encapsulation and the obtaining of large and heterogeneous particles that require further processing. Higher encapsulation efficiency can be ensured by the reverse-phase evaporation, solvent injection and dehydration–rehydration techniques. Typically, the liposomes require post-production treatment, e.g., sonication, extrusion and high-pressure homogenization. A more advanced method for obtaining liposomes is the microfluidic-based technique, which consists of the controlled mixing of the components and their flow through tubes with a defined diameter from tens to hundreds of micrometres. This technique enables precise control with respect to their size and size distribution. This method can be combined with in-line sample purification and drug loading to develop a fast, one-step process that produces high concentrations of reproducible drug-loaded liposomes with high efficiency [20,23,24,25]. An interesting type of liposomes are magnetoliposomes, which possess a magnetic core surrounded by a double lipid layer. Due to this structure, liposomes can be excited by magnetic radiation inducing local hyperthermia within the tumour [26,27]. There are numerous reviews focusing on trends in the design and use of liposomes for tumour targeting [20,24,25,28,29], comparison of liposomes and polymersomes [30,31] or localized delivery of liposomal formulations of drugs, which have been investigated pre-clinically and clinically in the last ten years [32].

Dendrimers (Figure 2C) can be made of various compounds, e.g., poly(propylene imine) (PPI), poly(L-lysine), poly(amidoamine) (PAMAM) and PAMAM-organosilicon (PAMM-OS). They possess a predictable shape, size, molecular weight and structure, which allows for the design of carriers with specific properties (e.g., lipophilicity, affecting the penetration through cell membranes) or the modification of adjustable ends, which allows to obtain dendrimers with specific targeting cancer cells and improved safety profile [33]. Dendrimers can increase the stability, solubility and bioavailability of active agents [34]. They can be used to deliver drugs, genes or contrast agents. Dendrimers are obtained as a result of a sequence of reactive steps through a step-by-step controlled synthesis related to molecular chemistry and polymer chemistry. A repeating structure of monomers is used, creating successive generations and an extensive structure of dendrimers. The synthesis of dendrimers involves divergent or convergent growth methods. In the convergent growth method, the synthesis commences with the component elements (branches), which are then combined into a single dendrimer structure. Due to the complexity of dendrimer synthesis processes and their associated costs and structural control problems, accelerated methods of dendrimer synthesis were developed, such as the double exponential growth technique, double-stage convergent method, hypercore approach, hypermonomer method or the branched monomer approach [33,35,36,37]. The properties, structure, synthesis and use of dendrimers were characterized in several recent review articles [35,38,39,40].

Polymeric micelles (Figure 2B) are small (50–200 nm), core-shell structures which ensure the extended circulation of drugs by avoiding uptake by the reticuloendothelial system. They are obtained from various kinds of amphiphilic polymers, e.g., copolymers of poly(ethylene glycol) (PEG), poly(lactide) (PLA) or poly(ε-caprolactone) (PCL) [41]. The micelles are composed of a hydrophilic shell and a hydrophobic core. Micelles obtained from amphiphilic block copolymers are more stable and ensure more versatility compared to lipid nanoparticles [42]. Surface modifications of polymeric micelles can also provide active targeting to cancer cells. Additionally, the release of drugs from micelles can be activated by the tumour microenvironment (pH, enzymes, temperature) or by external factors (e.g., heat, light, ultrasound and the magnetic field) [43,44,45]. Polymeric micelles are the most frequently obtained by means of three methods: direct dissolution, solvent evaporation or dialysis [46]. The choice of method for forming micelles from block copolymers depends on the solubility of the copolymer. If the copolymer is relatively water soluble, micelles can be formed by using the direct dissolution method, in which the copolymer is simply added to the aqueous media at a concentration above the critical micellar concentration and the drug is allowed to partition into the core of the micelles [47,48]. For more hydrophobic copolymers, the dialysis or film hydration methods can be utilized. Film hydration involves the dissolution of the copolymer and drug in a volatile solvent, which is then evaporated to leave a film in the bottom of a vial. Buffer or water is then added under heated conditions and agitated to dissolve the polymer film [49,50]. In dialysis, the copolymer and drug are solubilized in a water-miscible organic solvent followed by dialysis against aqueous media to remove the solvent, with the micelles being formed with the addition of water. The co-solvent evaporation procedure involves the dissolution of the copolymer in a volatile, non-water-miscible organic solvent, with this being added into a rapidly stirred aqueous media, with or without a surfactant, followed by the evaporation of the solvent [47,51]. In this technique, the drug may be loaded during the formation of micelles or via a two-step process when the drug is loaded into the aqueous micellar solution [52]. The recent advances in polymeric micelles have been described in numerous articles focused on their design, characterization and biological significance [53], anticancer drug delivery [54,55], the multimolecular interactions between block copolymers and the loaded drugs, proteins or nucleic acids [56], the stability of micelles [57], techniques for their characterization and assessment in biorelevant conditions [58] and the stimuli-sensitivity of micelles [59,60,61].

Metal NPs (Figure 2E) have also garnered specific attention due to their potential to serve as multipurpose agents. Metallic and magnetic NPs can be used in magnetic resonance imaging and photothermal therapy [62,63]. They are regarded as one of the smallest nanocarriers because their diameter usually does not exceed 50 nm. AuNPs can be obtained via chemical, physical and biological synthesis. Metallic NPs can be surface-functionalized with a drug, ligand, protein, peptide, antibody, contrast agent, enzyme or nucleotide, which offers a wide range of clinical possibilities. The great advantage of these nanocarriers is the ability to control drug release depending on environmental factors, e.g., pH. Gold, silver, iron and/or iron oxide, zinc, titanium, cerium oxide, nickel, copper, magnesium, barium, calcium and bismuth-based metal NPs have been reported for cancer treatment [62,64].

Solid lipid nanoparticles (SLNPs), which are characterized by a size of 50–500 nm, are obtained from lipids that remain solid at room temperature and at human body temperature. They are composed of a lipid monolayer enclosing a solid lipid core (Figure 2D). SLNPs are biodegradable, biocompatible and non-toxic. Two of the most popular methods of obtaining SLNCs are the hot homogenization technique and the solvent emulsification and diffusion method [65]. Although SLNPs can transport both hydrophobic and hydrophilic agents, they are especially useful in the delivery of hydrophobic drugs due to their high affinity to the core [25]. The feature that distinguishes SLNPs from non-lipid nanocarriers is their easy uptake by cells through the lipid bilayer. They are a good nanodevice for the delivery of inhaled and transdermal drugs [66,67].

A number of these delivery systems have already been approved by the US Food and Drug Administration (FDA) for the treatment of cancer in humans with various drugs. The examples include: a nanoparticle combination of Ptx with albumin (Abraxane^®^) [68], pegylated liposomes with Ptx (Caelyx^®^ (Europe), Doxil^®^ (US)) [69], non-pegylated liposomes with Ptx (Myocet^®^) [70] or a pegylated form of cytarabine used in the intrathecal treatment of meningitis in the course of lymphoma (DepoCyte^®^). However, there is still no effective delivery system for Dtx and Res registered as a medical product, although numerous clinical trials are being conducted.

## 3. Docetaxel

### 3.1. Docetaxel as an Anticancer Drug

Dtx is a chemotherapeutic agent which is used in the treatment of a variety of cancers in monotherapy and combination therapy. Dtx has demonstrated antitumour activity against recurrent ovarian cancer, non-small cell lung cancer, breast cancer, squamous cell carcinoma of the head and neck and gastric cancer [71].

**Figure 3:**
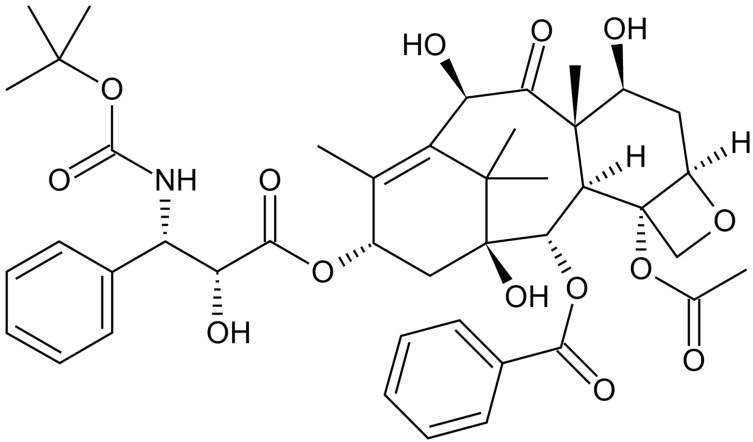
Chemical structure of docetaxel (Dtx).

Dtx (Figure 3) is a cytotoxic taxane, semisynthetic product obtained by the esterification of 10-deacetylbaccatin III, which has been isolated from the needles of *Taxus baccata* since 1981. After intravenous administration, Dtx has a large volume of distribution, binds to plasma proteins and is metabolized by the cytochrome CYP3A4 [72]. Dtx is an antimicrotubular agent that mainly exerts a cytotoxic effect by disrupting the microtubule network in cells that is essential for mitotic and interphase cellular functions (it supports and stabilizes the formation of microtubules and prevents the depolymerization of microtubules), thus inhibiting proper cell division [73].

Dtx toxicity and its systemic side effects are one of the serious problems that limit the effectiveness of treatment in humans. Neutropenia is one of the most common adverse reactions, occurring in 50–80% of patients treated with the basal dose of 100 mg/m^2^. In addition, hypersensitivity reactions (HSRs) characterized by hypotension, bronchospasm and/or generalized rash/erythema, skin toxicity associated with a risk of major infection, fluid retention, hypoalbuminemia and cardiac, renal or hepatic dysfunction are common. Dtx can also cause neurotoxicity (motor and sensory neuropathies: 40%), asthenia (60–70%), stomatitis and diarrhoea. Often, during therapy, it is necessary to reduce the Dtx dose to 30–75 mg/m^2^ (in 5–25%) due to toxicity, despite the use of premedication with corticosteroids (dexamethasone 8 mg, betamethasone 8 mg or prednisolone 50 mg) and the prophylactic use of granulocyte colony-stimulating factors (G-CSF) [73,74,75].

The second major problem associated with the effective action of Dtx against cancer cells is the emergence of treatment resistance, which results in a decrease in the anticancer effect. The first possible resistance mechanism is related to the altered expression of the β-tubulin isotype. Isotype I is comprised of 80–99% cellular β-tubulin in benign cells. On the other hand, an increase in the amount of the β-III isotype increases the dynamic instability of microtubules, impairs the rate of microtubule assembly and increases resistance to taxanes. The second Dtx resistance mechanism follows the classical pattern of MDR and may be reversible with appropriate treatment. Another source of Dtx resistance may be an increase in caveolin-1 expression, which is the main component of membrane vesicles involved in small molecule transmembrane transport and intracellular signalling [74].

### 3.2. Docetaxel Drug Delivery Systems

**Figure 4:**
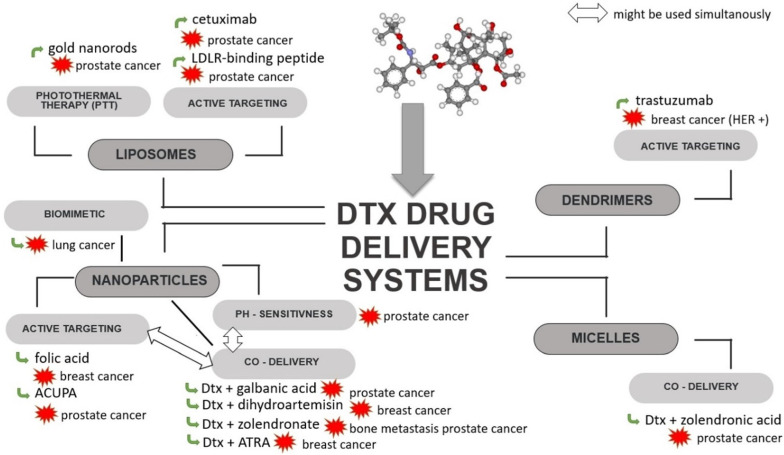
Scheme of strategies used for Dtx-loaded delivery systems. ACUPA—((S)-2-(3-((S)-5-amino-1-carboxypentyl) ureido) pentanedioic acid; ATRA—all-trans-retinoic acid; Dtx—docetaxel; HER—human epidermal growth factor receptor; LDLR—low-density lipoprotein receptor.

In order to overcome the limitations associated with Dtx, such as its dose-limited systemic toxicity, the effect of tumour drug resistance or the problem of delivering the therapeutic concentration directly to the tumour, efforts are being made to create an advanced delivery system targeting tumour cells. To achieve this goal, several kinds of Dtx carriers have been developed, such as liposomes [76,77,78,79], solid lipid nanoparticles (SLNPs) [80,81,82,83], dendrimers [84,85,86], nanoparticles (NPs) [87,88,89,90,91,92,93,94,95,96] and micelles [97] (Figure 4, Table 1), all of which will be described in this section.

Multifunctional liposomes have been developed for the delivery of Dtx and modified with RLT (low-density lipoprotein receptor (LDLR)—binding peptide), PEG and gold nanorods (GNRs). These liposomes can be used for combined chemo- and photo-thermal therapy (PTT) of cancer. Decoration with RLT allows for the active targeting of tumour cells with the overexpression of LDLRs, which is associated with an increased demand for fatty acids by rapidly dividing cells. With the use of GNRs and near-infrared radiation (NIR), which has ability to penetrate deep tissue, local heating occurs, causing cell damage and death as a result of necrosis or apoptosis [76].

Immunoliposomes targeted with epidermal growth factor receptor (EGFR) and loaded with Dtx have been tested against prostate cancer. Cetuximab, which was used to increase the cellular uptake of liposomes, is an anti-EGFR chimeric-murine monoclonal antibody, a biological drug that competitively binds to the EGFR on EGFR-positive cancer cells (e.g., DU145). It has been observed that cetuximab-modified liposomes show increased cellular uptake and cell selectivity compared to non-targeted liposomes [77]. Also, liposomes functionalized with transferrin (TF) developed for the delivery of Dtx may show advantages in the treatment of prostate cancer. An in vitro cytotoxicity test showed higher toxicity in TF-modified liposomes compared to unmodified nanoparticles. Moreover, modification with transferrin resulted in a slow and sustained release of Dtx, most likely through liposome stabilization [79]. Another example of Dtx immunoliposomes targeted by a surface monoclonal antibody are trastuzumab liposomes prepared to target breast cancer cells overexpressing HER-2 receptors. The increased efficacy of trastuzumab-functionalized and encapsulated Dtx liposomes compared to free Dtx and non-Dtx liposomes with trastuzumab was demonstrated against breast cancer cells (MDA-MB-453 and SKBR3) [105]. However, the potential of EGFR- and TF-functionalized liposomes require further study to confirm their effectiveness in vivo.

An example of dendrimers that actively target tumour cells is the system obtained by the conjugation of PAMAM with trastuzumab and Ptx or Dtx. The developed dendrimers showed high antitumour efficacy against SKBR-3 cells that are characterized by the overexpression of HER receptors and low toxicity against HER-negative tumour cells [86].

**Figure 5:**
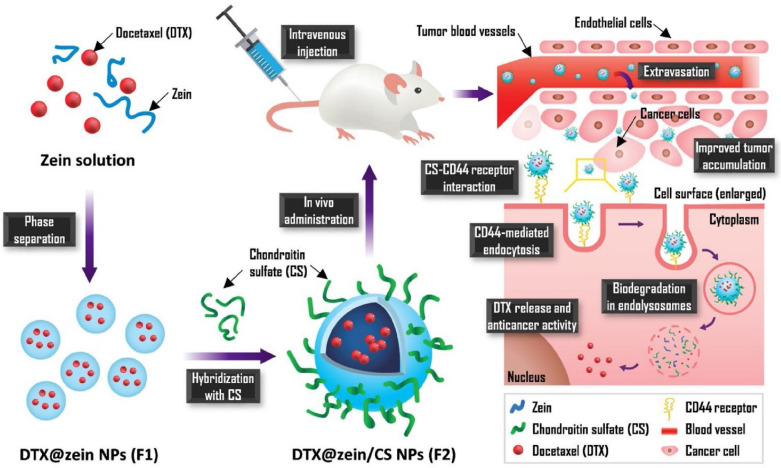
Scheme for the preparation Dtx-loaded CS-hybridized zein NPs and their mechanism of action. Reprinted with permission from Ref. [99]. Copyright (2021) Elsevier.

A recently published study on nanoparticles obtained from chondroitin sulphate (CS) and zein and loaded with Dtx indicated very favourable properties and antitumour effects in comparison to Taxotere^®^. The NPs enabled a 23% increase in cellular uptake and 35.5 times greater accumulation in the tumour. Zein/CS NPs were obtained using the solvent displacement method followed by CS hybridisation during the self-assembly process via electrostatic interaction. After intravenous administration, the NPs are expected to pass beyond the blood vessel and reach cancer cells due to interaction between CS and the CD44 receptor. Receptor-mediated endocytosis is responsible for improving cellular uptake efficiency. The mechanism of action of these NPs is shown in Figure 5 [99].

Dtx-loaded nanoparticles obtained from poly(lactide-co-glycolide) (PLGA) and modified with folic acid (FA) were developed by Poltavets et al. An in vitro study demonstrated the ability of the particles to overcome MDR and their higher cytotoxicity against cells overexpressing folic acid alpha receptor (FRα) compared to the free drug. The efficacy of these nanoparticles has also been confirmed in vivo [14]. Functionalization with folic and was also used in gold nanoparticles (Au-NPs) coupled to Dtx. An in vitro study of Au/NPs/FA/Dtx demonstrated that such particles achieve 50% higher cytotoxicity compared to free Dtx [98].

Biomimetic nanoparticles developed for the targeted delivery of Dtx to lung cancer combined the latest advances in nanomedicine with naturally occurring cell membranes to increase the antitumour activity of the cytostatic drug. They were obtained by the incorporation of PLGA NPs loaded with Dtx into platelet membrane (PM). Coating the surface of nanoparticles with PM is advantageous because of their subsequent immune escape ability and cancer targeting properties, resulting in enhanced tumour growth suppression. An in vitro study demonstrated that PM-coated NPs loaded with Dtx have an ability to inhibit cell growth at a concentration range of 0.31–10.00 μg/mL, which was also confirmed in vivo [104].

#### 3.2.1. Carriers for Co-Delivery of Dtx with Another Drug

In addition to the delivery strategies of Dtx described above, more advanced delivery systems have also been developed, e.g., ligand-targeted NPs for the co-delivery of Dtx and another anticancer agent. An example are NPs co-loaded with Dtx and galbanic acid (Gba) for targeting prostate-specific membrane antigen (PSMA)-positive cancer cells. The NPs were obtained from poly(lactide)-co-poly(ethylene glycol) (PLA-PEG) modified with ((S)-2-(3-((S)-5-amino-1-carboxypentyl)ureido) pentanedioic acid (ACUPA), which is an inhibitor targeting PSMA [103]. It should be noted that similar polymer nanoparticle systems, including those targeting PSMA, have been the subject of promising clinical trials, as described in Section 3.2.1.

pH-sensitive NPs for the simultaneous delivery of Dtx and dihydroartemisin (Dha) have been investigated for the treatment of transient breast cancer. The mechanism of synergistic action of Dtx with Dha was explained by induction of apoptosis mediated by mitochondria. As a result of the simultaneous action of both active agents, p53 expression and ROS levels are increased and the release of cytochrome C to the cytoplasm is promoted, which activates caspase 3 [102]. Another pH-sensitive nanoparticle developed for the co-delivery of zoledronate (Zol) and Dtx are bone-targeted calcium phosphate-polymer hybrid NPs, which were tested against prostate cancer bone metastasis. CaP (calcium phosphate) was used to obtain the pH-sensitivity of the micelles. Good antitumour properties were confirmed in the nude mice model because the use of high doses of Dtx/CaP/HP caused more a significant decrease in tumour volume compared to free Dtx and Zol [101].

A co-delivery system for Dtx and all-trans-retinoic acid (ATRA) was obtained from the poly(β-amino ester) (PBAE). ATRA, a derivative of vitamin A, is effective against some cancer cell lines; however, due to its low water solubility, low plasma concentration and side effects, its clinical use has been limited. Moreover, it was found that in combination with traditional chemotherapeutic agents, ATRA exhibits antitumour activity against metastatic breast cancer. An in vitro study of ATRA-PBAE-NPs revealed not only a successful pH sensitive drug release and the synergistic cytotoxic activity of ATRA and Dtx but also their antiangiogenic properties [100].

#### 3.2.2. Clinical Trials of Docetaxel Nanoparticles

Treatment with Dtx has been the subject of numerous clinical trials and more than 1700 of these can be found in the ClinicalTrials.gov (provided by the US National Library of Medicine) register. Among the many research studies of the use of the traditional intravenous form of Dtx, there are several clinical trials that have evaluated NPs as delivery systems of Dtx: five clinical studies involving the use of the BIND-014, three testing results of LE-DT treatment, one regarding the application of Dtx-PNP (a polymeric nanoformulation of Dtx) and two evaluating Cripec (Table 2). LE-DT is a liposome-entrapped Dtx designed by NeoPharm and studied for the treatment of solid tumours (pancreatic cancer and prostate cancer). A phase one study (NCT01151384) of LE-DT showed that the use of this drug formulation had clinical benefits for 41% of patients [106]. Phase two studies were launched, and one of them, on metastasis in pancreatic cancer, was completed in 2012, with the second one, which was withdrawn in 2010, testing LE-DT in prostate cancer. Another example of a carrier developed for Dtx that has reached phase two is BIND-014, which is composed of Dtx encapsulated in a polymer core made of a hydrophobic poly(lactide) surrounded by a hydrophilic poly(ethylene glycol) conjugated with a small molecule of PSMA-targeting ligands. In the first phase of clinical trials, BIND-014 showed satisfactory properties. In NCT01300533, a clinical study was conducted in 52 patients with various types of neoplasms to determine the pharmacokinetics, safety profile and antitumour activity of BIND-014. It was observed that the BIND-014 was well tolerated by patients, had dose-dependent linear pharmacokinetics and a prolonged circulation time. The BIND-014 accumulated in a variety of cancer cells independently of PSMA detection. However, due to difficulties in phase two related to a low response to treatment (NCT02479178) and other factors related to the manufacturer, this research on BIND-014 was suspended [107,108].

Another example of polymeric nanoformulation for the delivery of Dtx is Dtx-PNP, which has been tested preclinically in the orthotopic mouse model and was shown to be more effective in inhibiting tumourigenesis than free Dtx or gemcitabine. The NCT01103791 study was conducted in 19 patients with various types of cancer (e.g., colon, rectum, cervix, breast, bladder, kidney, pancreas cancer) and in various doses ranging from 20 to 75 mg/m^2^. The goal of this study was to find the maximum tolerated dose (MTD) and dose limiting toxicity (DLT). The maximum dose of Dtx-PNP was found to be 75 mg/m^2^, despite the fact that all patients developed a symptom of neutropenia, which was normalized within 7 days. In addition, it was found that the partial remission was at the level of 22%. However, these clinical trials involved a small control group, and further research is necessary [87]. Recently, in December 2020, a phase two clinical trial (NCT03742713) of Cripec^®^ in ovarian cancer treatment was completed; however, the results have not yet been presented. Cripec^®^ is novel formulation that consists of Dtx-entrapped core-cross-linked polymeric micelles (Dtx-CCL-PMs). It was developed by Cristal Therapeutics via the conjugation of Dtx to linker and subsequent covering with polymer to achieve a stable nanoparticle. An in vitro and in vivo study showed that Cripec^®^ is more safe and efficient than free Dtx in breast cancer treatment [109,110].

### 3.3. Summary

Dtx is a semisynthetic anticancer mitotic chemotherapy drug used against a wide range of solid tumours, including breast, lung, head, prostate, neck, non-small cell lung and ovarian cancer. However, due to the limitations of Dtx, such as its low water solubility, systemic toxicity and severe allergic reactions, various kinds of NPs have been formulated for Dtx delivery, such as liposomes, dendrimers, micelles and SLNPs (Table 1). The achievements of the last 10 years clearly demonstrate the prevalence of advanced systems designed for the targeted delivery of Dtx. For this purpose, different kinds of ligands have been tested, e.g., RLT, TF and EGFR, and these tests demonstrated that functionalized NPs have a higher efficiency than non-targeted delivery systems. Another direction of the research has been focused on the development NPs suitable for the delivery of combinations of docetaxel and other drugs (e.g., Gba, Dha, Zol and ATRA), and these have exhibited enhanced cytotoxicity against cancer cells compared to the single drug. The effectiveness of the carriers of Dtx and Dtx with other drugs developed to date is frequently supported by solid experimental data obtained from in vitro and in vivo studies. It is very promising that some of the NPs have already reached the clinical study stage (Table 2); however, their number is still very limited.

## 4. Resveratrol

### 4.1. Resveratrol as an Active Agent

Res (trans-3,4′,5,-trihydroxystilbene) is a natural polyphenolic molecule. This polyphenolic stilbene structure has two aromatic rings connected by an ethylene bridge and three hydroxyl groups [111,112].

**Figure 6:**
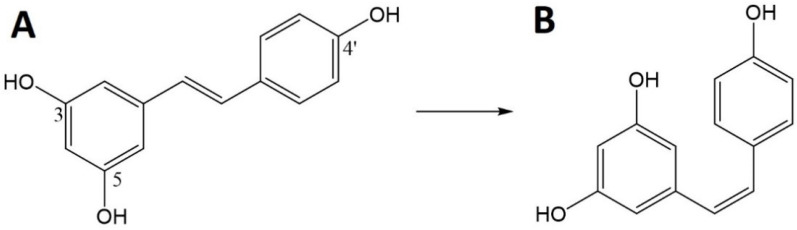
Chemical structure of resveratrol (Res)—*trans* (**A**) and *cis* (**B**).

Res can exist in the form of two isomers (Figure 6), *cis* and *trans*. In nature, the *trans* isomer is common and the less stable *cis* isomer is formed from *trans*-Res by the action of light, UV and heat [113]. However, even the more stable trans-isomer can be easily degraded by numerous factors, both physical and chemical, including temperatures above 37 °C, pH above 6.8 and exposure to light [112]. Res is also classified as a phytoestrogen because it can bind to estrogen receptors [111]. Historically, Res has been associated with Asian medicine and red wine, and then linked with the so-called the French paradox that cardiovascular diseases and hypercholesterolemia were less frequent in the French population. It has now been found that Res is present in many plants, e.g., grapes (*Vitis vinifera*), mulberries (*Morus* sp.) and peanuts (*Arachis hypogaea*) [112,114]. Moreover, research confirms the presence of its high content in the *Itadorii* plant and tea [115].

Numerous studies confirm the chemoprotective [115,116], chemopreventive [117,118,119] and chemotherapeutic properties of Res. The chemotherapeutic effect of Res covers a broad spectrum of therapeutic effects such as antiviral [120], antifungal and antibacterial [121], antiaging, antioxidant and anti-inflammatory activity [122]. Additionally, it has a cardioprotective and analgesic effect [123] or can be used as an antiobesity agent [124]. Res also plays a role in the treatment of neurological diseases, such as Alzheimer’s [125] or Parkinson’s [126].

**Figure 7:**
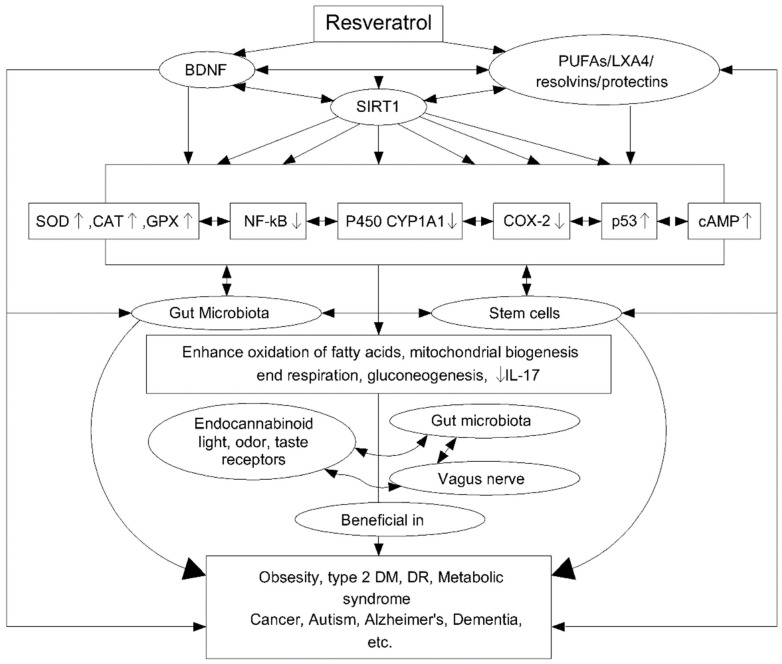
Scheme of resveratrol (Res) cellular action and its further impact. Reprinted and adapted with permission from Ref. [127]. Copyright (2020) Elsevier.

The antioxidant properties of Res are related to the ability to regulate many signalling pathways (Figure 7). Studies have been carried out on peripheral blood mononuclear cells (PBMC) to assess the antioxidant properties of Res under oxidative stress in the cells of middle-aged and elderly people. The Res resulted in a significant reduction in reactive oxygen species (ROS) in both age groups, but with a better performance in the middle-aged group (92.6%) than in the older group (76.7%). The effect of Res on signal pathways was also studied, finding an effect not only on AMP-activated protein kinase (AMPK) and the sirtuins signalling pathway (SIRT1) but also on p38-Mitogen activated protein kinase (MAPK) and PKA and AkT/PKB pathways [122]. In turn, the anti-inflammatory properties of Res are associated with the regulation of cytochrome P450 (CYP1A1), p53, cyclooxygenase enzymes (COX), transcription factor NF-kB, Fas/Fas ligand mediated apoptosis, mTOR and cyclins, various phosphor-diesterases which have an impact on cAMP level and modulate the activity of the Epac1/CaMKKb/AMPK/SIRT1/PGC-1a pathway. This, in turn, affects the oxidation of fatty acids, mitochondrial respiration and their biogenesis and gluconeogenesis. The anti-inflammatory effect of Res is additionally related to its ability to trigger apoptosis in activating T lymphocytes and to inhibit tumour necrosis factor (TNF-α), interleukin-17 (IL-17), the expression of hypoxia-induced factor (HIF-1α) and vascular endothelial growth factor (VEGF) [127].

Furthermore, several reports have indicated potential antiproliferative or anticancer effects of Res per se against many types of human cancers [128], and the first report mention its potential anticancer activity was published in 1997 [129]. The cancer preventive effect of Res has mainly been attributed to its antioxidant and anti-inflammatory/immunoregulatory profiles. It has been reported that Res can downregulate the expression of multidrug-resistant genes encoded for P-gp and inhibit the mammalian target of rapamycin via pyruvate kinase isoenzyme type M2, thereby preventing cancer cell metabolism. Res has shown promising cytotoxic effects on a wide range of solid tumour cells, e.g., breast, liver, prostate, colorectum, pancreas, lung and gliomas [111,114,115].

However, despite high interest in Res as an anticancer drug, the complete mechanism of anticancer activity has not been elucidated and research is continuing. A cytotoxicity study of Res was performed in vitro on melanoma lung metastasis cells and in vivo on a mice model. The results showed that the number of melanoma lung metastasis cell colonies in mice treated with Res was significantly lower. Moreover, 70% of the Res-treated animals survived for 1 month, while all the controls were dead by then [130].

The cytotoxicity of Res has also been proved also in a study of cell cycle arrest and inducing apoptosis of breast cancer cells. Transcriptome and qRT-PCR data showed that among 330 assessed genes, 103 were upregulated after the administration of Res and 227 were downregulated. In addition, Res has been shown to arrest cells in the S phase, to reduce proliferation and to induce apoptosis. The process was dose- and time-dependent [131]. The effect of modulated electro-hyperthermia (mEHT) with the administration of Res and curcumin (Cur) via colorectal cancer as well as the impact of such a procedure on the cell cycle was also studied. By using mEHT and disrupting the cell membrane structure, the cellular uptake of Res and Cur increased, causing cell cycle arrest and the apoptosis of CT26 cells. Moreover, CT26 tumour growth in BALB/c mice was significantly inhibited [132].

### 4.2. RES-Loaded Nanoparticles

Despite its broad healing properties, Res has several disadvantages that make it difficult to use in medicine. First of all, it is poorly soluble in water and decomposes quickly under the influence of light, oxygen and oxidative enzymes. Due to the fact that it is a sensitive molecule, the *trans* isomer is easily converted into *cis*—with much weaker activity. Another obstacle is the rapid metabolism and elimination of Res and its conjugation with sulphates and glucuronic acid, which prevents it from exerting its effect. All the above-mentioned features result in the low bioavailability of Res and mean that its administration does not bring about the expected therapeutic effects [18]. This is the reason behind the study of the development of delivery systems for Res that will improve its biodistribution. The encapsulation of Res in NPs enables it to be protected from decomposition by physicochemical factors, increase its uptake by cells as well as ensure the controlled site or time of release from the carrier, and, thus, this increases the bioavailability of Res. Therefore, this part of the review focuses on the presentation of Res-loaded NPs tested for cancer treatment (Table 3).

The dosage form that can protect Res from a loss in stability and improve its antioxidant activity may be zein NPs made from a combination of bovine serum albumin (BSA) and conjugates of BSA and caffeic acid (CA). The increase in the stability and antioxidant activity of Res encapsulated in zein-BSA NPs and zein-BSA-CA NPs was observed in comparison to free Res and Res-loaded zein-NPs, suggesting that the developed NPs may be used in the food industry for effective Res supplementation [142].

For medical use, to enhance the low bioavailability (<1%), solubility and stability of Res, Res-loaded NPs were obtained from chitosan (CS) and poly(γ-glutamic acid) (γ-PGA) (Res-chitosan/γ-PGA NPs) using the ionic gelation method. The increased uptake of Res-CS/γ-PGA NPs by colon carcinoma cells in comparison to non-encapsulated Res was observed [143].

Res-loaded gold NPs (Res-AuNPs) were developed and studied for breast cancer treatment. It was found that the level of matrix metalloproteinases (MMPs) and cyclooxygenase-2 (COX-2) facilitating tumour angiogenesis as well as the nuclear transcription factor-κB (NF-κB) and activator protein-1 (AP-1) that promote cancer invasiveness through gene regulation were lowered after the use of Res-AuNPs [133].

Res-loaded NPs have been also developed from poly(ε-caprolactone) (PCL) and tested against murine melanoma. The results of an in vivo study showed that Res-encapsulated PCL-NPs reduced the volume of tumours significantly compared to the control group and free Res. Moreover, they increased necrosis and inflammation in the cancer area [134]. Also, poly(lactide-co-glycolide) (PLGA) was used to produce Res-loaded NPs. The chemopreventive effect of Res-PLGA NPs on prostate cancer cells was investigated. Although the chemopreventive properties of free Res on tumour cells has been the subject of clinical trials, a satisfactory effect has not been achieved, probably due to the poor bioavailability, solubility and stability of Res. As expected, the encapsulation of Res in PLGA resulted in the improved availability of Res, which exhibited cytotoxicity against prostate cancer cells without any undesirable effects on normal cells, even at high doses of Res [136].

Res-loaded SLNPs (Res-SLNPs) were analysed for the delivery of Res to human breast cancer. An in vitro study showed a greater number of the tested cells retained in the G0/G1 phase compared to free Res and reduced cell migration and invasion. In addition, the Res-SLNPs increased the expression level of Bax and decreased the level of Bcl-2, which plays important role in apoptosis [140].

FA-conjugated NPs obtained from HSA and loaded with Res were prepared and evaluated under in vitro and in vivo conditions. It was confirmed that FA-HSA-Res NPs have the ability to continuously and slowly release the drug. Compared to unmodified Res, FA-HSA-Res NPs increased the bioavailability of Res and inhibited the proliferation of neoplastic cells to a greater degree, which was also confirmed in vivo [138].

### 4.3. Resveratrol Clinical Trials

Res is the subject of numerous clinical trials in a wide variety of diseases, including cancer. The NCT00256334 phase one study of the effectiveness of Res in the treatment of colon cancer has already been completed. The 3 years-trial involved 11 patients. It was shown that oral Res supplementation may play a role in the prevention of colon cancer in humans [144]. The subsequent phase one study NCT00578396 on excised colorectal tumour cell lines involved the administration of mitomycin C and Res. These studies have demonstrated the increased upregulation of p21 (WAF1/CIP1) in cells treated with both drugs compared to cells treated with only one active agent. Two other clinical trials (phase one) on colorectal cancer (NCT00920803 and NCT00433576) have been conducted; however, the post-study information for these studies is poor [145]. The FDA has also registered a clinical trial for gastrointestinal tumours (NCT01476592). During this trial, the influence of Res on notch-1-signaling, effect on patient treatment and patient toleration on Res administration was investigated. It was shown that the administration of Res increased cleaved caspase-3 level in cancerous tissue, which means that the apoptosis in cancer cells was higher [145]. In phase two of the NCT00920556 clinical trial, a Res with bortezomib treatment for multiple myeloma was tested. The 24 patients involved in this study received 5 g of SRT501 (Res) orally daily before breakfast on 20 days of a 21-day chemotherapy cycle for 12 months. However, the obtained data showed an unacceptable safety profile, with no improvement in treatment effectiveness [146]. Another clinical study (NCT01370889) was focused on the effects of Res on the endocrine management of sex hormones in postmenopausal women with breast cancer and obesity (BMI > kg/m^2^). Measurements of the levels of estradiol, estrone and testosterone in the blood showed no changes, but the administration of Res caused a 10% increase in the concentration of sex steroid hormone-binding globulin (SHBG) [147]. It is worth noting that many studies are being conducted not only with respect to the treatment of disease entities but also in order to investigate the preventive effect of using Res or products containing Res, including in children.

### 4.4. Summary

Among active natural compounds, polyphenols are becoming highly popular because of their antioxidant, anti-inflammatory, antiaging and anticancer properties. Res is a polyphenolic stilbene derivative that has been shown to have promising cytotoxic effects on a wide range of solid tumour cells, e.g., breast, liver, prostate, colorectum, pancreas, lung and gliomas. However, due to the poor solubility of Res in water, its decomposition under the influence of light, oxygen and oxidative enzymes and the rapid metabolism and elimination of Res, more effective delivery systems are being developed. The several kinds of carriers have been studied for the encapsulation of Res include Res-zein-NPs, AuNPs, PCL-NPs, PLGA NPs, SLNPs and FA-HSA NPs (Table 3). Although most of them have been limited to in vitro study, it has been observed that the encapsulation of Res in NPs enables its protection from decomposition by physicochemical factors, an increase its uptake by cells as well as the controlled site or time of release from the carrier to be ensured, and, thus, the bioavailability of Res can be increased.

## 5. Co-Delivery of Docetaxel and Resveratrol

It is considered that co-encapsulation of a cytostatic drug with a natural chemosensitizer in the same delivery system can be an effective tool against cancer. This concept may not only combine the healing properties of two active agents but also enable a synergistic effect of action. The beneficial effect of the simultaneous use of a chemotherapeutic agent with a chemosensitizer is a reduction in the MDR effect, which often adversely affects the effectiveness of traditional chemotherapy. On the other hand, a wide range of expected properties, e.g., enhanced bioavailability, protection against biotransformation and controlled release or active targeting can be obtained by using the appropriate materials to form NPs. Moreover, using natural compounds in drug delivery systems may be one of the ways to overcome high costs and the time involved to undertake new drug research.

The aim of this part of the review was to explain the synergistic effects of Res and Dtx and to present the latest nanomedical achievements, in which NPs for the simultaneous delivery of these drugs have been developed.

### 5.1. Synergistic Effect of Docetaxel with Resveratrol

One of the earliest in vivo analyses of the pharmacokinetics of simultaneously administered trans-Res and Dtx was conducted in 2009 in mice implanted with a Dtx-resistant prostate carcinoma cell line (DU-145). Dtx and Res were administrated subcutaneously via Alzet osmotic pumps for 1 month. Data showed enhanced tumour regression and lower tumour angiogenesis compared to the control group treated only with Dtx [148]. Subsequently, it was found that Res can chemosensitize HER-2 positive breast cancer cells for Dtx action and overcome cancer resistance for its treatment by affecting the Akt axis. It was assessed that HER-2 overexpression through the activation of PI3K/Akt and upregulation of survivin allows cancer cells to escape from cytotoxic effects, e.g., Dtx through early mitotic exit and by activating the multi-drug efflux pump [149].

Further studies showed that Res enhances the cytotoxic properties of Dtx by increasing its intracellular level due to P-gp inhibition and the downregulation of MDR1 [71,128]. The combination of Res and Dtx up-regulates pro-apoptotic genes, provides a synergistic anticancer effect and generates an enhanced cancer treatment effect compared to the single drug. Dtx in combination with Res causes the significant downregulation of BCL-2, BCL-XL and MCL-1 (anti-apoptotic markers), upregulates BAX, BAK and BID (apoptotic markers) and cleaves PARP. Furthermore, the combination of Dtx and Res suppresses the expression of NF-kB p65, which increase the level of inflammatory markers, e.g., COX-2. Moreover, Dtx with Res decreases the levels of survivin protein, part of the inhibitor of apoptosis (IAP) protein family. It is found that a higher survivin level occurs in most cancers, with blocking the cell cycle arrest and restraining apoptosis by binding to caspase 3 and 7 [150,151]. In PCa cells, the combination of Dtx and Res has been shown to block the cell cycle by influencing key regulators and promoting apoptosis. The interaction was found to occur through a p53-dependent and p53-independent mechanism. In turn, the p53 protein is a known inhibitor of CDKs and affects the p21^WAF1/CIP1^ and p27^KIP^ pathways, which play a significant role in apoptosis by stimulating pro-apoptotic proteins and inhibiting those that block apoptosis [150]. Additionally, one of the newest findings indicated that a combination of Res and Dtx has also an effect on ROS-induced mitochondrial dysfunction and DNA damage [152].

Nevertheless, an explanation for the mechanism of synergistic action of Dtx and Res is still under investigation and has not yet been fully elucidated. Regardless of this, their synergistic effect has been confirmed and is reflected in the increased toxicity of Dtx administered with Res, in comparison to the effect of free Dtx, as well as in the fact that the co-administration of Dtx with Res enables to overcome cell drug resistance, leading to cell death.

### 5.2. Nanoparticles for Co-Delivery of Docetaxel and Resveratrol

The co-encapsulation of several drugs into the same delivery system enables their simultaneous intracellular release and action. The co-administration of two or more pharmacologically active agents with different mechanisms of action (combination therapy) is recognized as more efficient compared to conventional therapy based on a single therapeutic agent [153,154,155].

Several studies have been carried out to evaluate the synergistic effects of Res and Dtx loaded in a nanoparticle delivery system (Table 4). The effect of Res and Dtx co-encapsulated in polymeric micelles prepared from methoxyl poly(ethylene glycol)-poly(D,L-lactide) (mPEG-PDLA) was analysed. It was found that the 1:1 ratio of Dtx and Res is the most favourable for the synergistic effect of both compounds on MCF-7 cells. The loading properties of Dtx and Res were comparable, and their release was rapid for the first 12 h (80% of drugs released until 72 h). The cytotoxic effect of micelles loaded with Dtx and Res was significantly higher compared to free drugs and single-drug loaded micelles [71].

Planetary ball milled (PBM) NPs functionalized with folic acid and co-loaded with Dtx and Res were developed for the treatment of drug resistance in prostate cancer cells. After preparation in a milling jar, the NPs containing Dtx and Res were coated with a FA-PCL-PEG copolymer. This innovative technique for the formation of nanoparticles loaded with a cytotoxic drug has been described in US Patent 8,231,907. This method uses a milling jar containing heat-absorbent zirconium oxide planetary milling balls. The milling balls rotate about their own axis and in the opposite direction around the common axis of the chamber wheel. Through the appropriate use of controlled centrifugal forces as well as the regulation of the length and number of cycles and the number of zirconium oxide balls, NPs with the desired properties are created [151,157]. Cytotoxicity and cell viability was tested on PC3 cells and a cell line resistant to Dtx (PC3-R). Time and dose-dependent Dtx re-sensitization after the administration of FA-Dtx+Res-PBM NPs was observed. The IC50 of Dtx and Res was reduced to a greater degree after treatment with FA-Dtx + Res-PBM NPs compared to free drugs [151].

Another ligand used to target NPs co-loaded with Dtx and Res is EGFR. Core-shell lipid-polymer hybrid NPs (LPNs) conjugated to EGFR were developed for the treatment of non-small cell lung cancer (NSCLC). LPNs are considered to be appropriate carriers for creating complex targeted delivery systems due to the fact that they combine the biosafety of liposomes and the versatility of polymeric materials. An in vitro study showed that the cellular uptake of EGFR conjugated LNPs was higher in HCC827 cells with a positive mutation (EGFR M+). The EGFR-RES+Dtx-LPNs exhibited a higher level of efficiency than single drug loaded NPs, which proves the synergistic effect of Dtx and Res. Also, in vivo tests revealed a the greater accumulation of EGFR-Res+Dtx–LPNs in neoplastic and lung tissues [156].

The results of the studies described above are promising; however, their cytostatic potential, efficiency and therapeutic effects require further analysis and, subsequently, confirmation in clinical trials.

### 5.3. Summary

It is considered that the co-encapsulation of cytostatic drug with natural chemosensitizer in the same delivery system can be effective tool against cancer. The synergistic effect of Dtx and Res has been confirmed and is reflected in the increased toxicity of Dtx administered with Res in comparison to the effect of free Dtx. Moreover, the beneficial effect of the simultaneous administration of Dtx as a chemotherapeutic agent with a chemosensitizer (Res) enables the MDR effect, which often adversely affects the effectiveness of traditional chemotherapy, to be overcome. These encouraging results are the driving force behind the development of nanocarriers for the co-delivery of Dtx and Res. This is a novel approach, so there are only few published reports on co-delivery systems for Dtx and Res (Table 4). The results show the synergistic effect of the drugs, which provides a rationale for further research.

## 6. Challenges and Opportunities

The use of NPs as anticancer drug delivery systems is currently one of the most important research areas in cancer treatment and provide promising alternatives to the limitations of conventional chemotherapy. According to the presented results, NPs may improve biodistribution, enhance cytotoxicity against cancer cells, reduce the side effects of commonly used cytostatic drugs, such as Dtx, and may be also used for active agents of natural origin, such as Res. The increasing interest in using the combination of Res as a chemosensitizer with Dtx (cytostatic drug) for overcoming cancer resistance to treatment has also become a driving force behind the development of their delivery systems. The presented literature published within last decade shows great progress in the development of nano-delivery systems for Dtx and Res. Although the developed strategies have become more and more advanced, further effort is required in the development of a carrier, which, in addition to providing satisfactory results in laboratory tests, would also allow for clinical translation. The data published data so far on carriers enabling the co-delivery of Dtx and Res are limited. This subject is expected to be explored in the near future. Several problems require special attention, such as the development optimal nanocarriers for co-loading Dtx and Res and the verification of their usefulness under in vitro and in vivo environments involving various kinds of cancers. The development of an optimal nanocarrier may present a significant challenge, especially taking into account that drugs should be encapsulated according to the particular ratio that shows the highest synergistic effect and that drugs may not have the same affinity for loading into the carrier. Different factors, such as drug chemistry or molar mass, may affect a drug’s encapsulation efficiency. Polymeric drug delivery systems should be characterized comprehensively, including related inter- and intramolecular interactions. It is commonly described in the literature that polymer–drug interactions or drug–drug interactions influence the rate of drug diffusion and the degradation of kinetics [158,159].

Undoubtedly, finding a system that might respond to intra- and inter-tumoural variability remains a significant challenge. Neoplasms have a large variety of microenvironments, and there is significant heterogeneity with respect to the molecular, pathologic and clinical features of each tumour type, both of which present huge challenges for modern medicine.

## 7. Conclusions

Cancer remains one of the most common causes of death in the world. However, the effectiveness of the available treatments is still limited. Dtx is a chemotherapeutic agent commonly used in the treatment of a variety of cancers in monotherapy and combination therapy. However, its toxicity and systemic side effects are one of the serious problems that limit the effectiveness of treatment in humans. Therefore, various kinds of NPs have been studied for improving the bioavailability of Dtx and its therapeutic effects. Also, there is an increasing interest in using combinations of Dtx with other drugs to obtain a synergistic effect or chemosensitization. One of the combinations with beneficial therapeutic effects is the use of Res with Dtx. Res presents antioxidant, antiinflammatory and chemopreventive properties, but also limits multidrug resistance against Dtx, which is one of the main causes of failure in cancer therapy with this drug. However, the use of both drugs presents challenges, including poor bioavailability, the unfavourable pharmacokinetics and chemical instability of Res and the poor water solubility and dose limiting toxicity of Dtx. In order to overcome these difficulties, attempts have been made to create different forms of delivery for both agents. The review has presented the progress achieved in this regard in the last 10 years in delivery systems developed for the administration of a single drug (Dtx or Res) and the co-administration of these two active agents. It has been presented that various kinds of carriers have been considered for the delivery of Dtx and Res, such as liposomes, micelles, dendrimers, SLNPs and metallic NPs. The published data indicate the high efficiency of advanced NPs, which, in addition to their drug release effect, possess additional properties, e.g., pH sensitivity or active targeting abilities, and this line of research is expected to dominate in future. The published results indicate that the selective surface modifications of the carriers are highly effective in cancer chemotherapy. It has been shown that controlled delivery systems containing a single drug (Dtx or Res) or combinations of two drugs present greater efficiency in cancer treatment compared to free drugs. Also, treatment with a combination of two drugs with a synergistic effect presents higher cytotoxicity against cancer cells than a single drug. Based on the presented literature, it can be concluded that the co-delivery of Dtx and Res in an actively targeted transport system providing the simultaneous release of both drugs in cancer cells has a chance to fulfil the requirements of effective anticancer therapy and reduce limitations in therapy caused by MDR. Thus, there is a rationale for continued research on the development of advanced nanocarriers based on the active targeting of cancer cells by ligand-receptor recognition.

## Figures and Tables

**Table 1:** Examples of Dtx–loaded delivery systems for anticancer therapy.

DDS	Material	Size [nm]	EE [%]	Drug	Preparation Method	Location	Status	Ref.
GNRs/liposomes	DSPE-PEG_2000,_ CHOL, SPC, HSPC, RLT, PEG	163.15 ± 1.83	98.45 ± 0.37	Dtx	Film hydration	Prostate	In vitro-PC-3 cells In vivo-mice	[76]
Liposomes	DSPE-PEG_2000_, CHOL, SPC, cetuximab	67.47 ± 4.32	99.95	Dtx	Film hydration	Prostate	In vitro–PC-3, DU145 *cells*	[77]
Liposomes	DSPE-PEG_2000_, CHOL, SPC, transferrin	220.23 ± 3.95	37 ± 3.15	Dtx	Film hydration	Prostate	In vitro–PC-3, PNT2 *cells*	[79]
Dendrimers	PAMAM	n/d	n/d	Dtx/Ptx	Covalent method	Breast	In vitro–SKBR-3 cells	[86]
NPs	PLGA, FA	227.6 ± 5.9	n/d	Dtx	Solvent-evaporation	Breast	In vitro–HeLa, MCF-7 cells In vivo–mice	[14]
NPs	Au	18	n/d	Dtx	Chemical reduction	Lung	In vitro–H520 cells	[98]
NPs	GX1, PEG, DA, DGC	150.9 ± 3.5	52.7 ± 4.4	Dtx	Dialysis	Gastric	In vitro–co-HUVEC In vivo-mice	[91]
NPs	PCL, Pluronic F108	216 ± 3.4	86.0 ± 3.9	Dtx	Nanoprecipitation	Breast	In vitro–BT-474 cells In vivo–mice	[92]
NPs	Albumin, aptamer	62 ± 0.6	90.0 ± 0.7	Dtx	Salting-out method	Colon	In vitro–CT26 cells In vivo–mice	[93]
SLNPs	Span 80, Pluronic F127	128 ± 2.2	86.0 ± 2.4	Dtx	N/d	Breast	In vitro–4T1 cells In vivo–mice	[82]
NPs	Chondroitin sulphate, zein	157.8 ± 3.6	64.2 ± 1.9	Dtx	Solvent displacement	Prostate	In vitro–PC-3 cells In vivo-mice	[99]
NPs	fluorescein-labelled wheat germ agglutinin (fWGA)-conjugated disulfide cross-linked sodium alginate	289	17.8	Dtx	N/d	Colon	In vitro–HT-29 cells	[88]
Synthetic high-density lipoprotein nanoparticles	Egg sphingomyelin (eSM), apolipoprotein A-1 mimetic peptide 22A	11.3	n/d	Dtx + Cho-CpG	Co-lyophilisation	Colon	In vitro–MC-38 cells In vivo-mice	[94]
NPs	PBAE	137.9 ± 2.09	20.36 ± 0.01	Dtx + ATRA	Solvent displacement	Breast	In vitro-HUVEC and MCF-7 cells	[100]
micelles	Cap, HA, PLA	144	n/d	Dtx + Zol	Dialysis	Prostate	In vitro–PC-3 cells In vivo-mice	[101]
NPs	PEG	153.1	n/d	Dtx + Dha	Dialysis	Breast	In vitro–4T1 cells In vivo-mice	[102]
NPs	H1 nanopolymer (folate–-polyethylenimine600–cyclodextrin)	117 ± 12.9	n/d	Dtx + dbait	N/d	Prostate	In vitro–CRPC, PC-3, DU145, LNCaP cells In vivo-mice	[96]
NPs	PEG, PLA, ACUPA	135 ± 15	45 ± 5	Dtx + Gba	Solvent-evaporation	Prostate	In vitro–PC-3, LNCaP cells	[103]
NPs	PLGA, PM	98.2	92.4	Dtx	Dialysis	Lung cancer	In vitro–A549 cells In vivo-mice	[104]

ACUPA—((S)-2-(3-((S)-5-amino-1-carboxypentyl) ureido) pentanedioic acid; Au—gold; Cap—calcium phosphate; CHOL—cholesterol; cho-CpG-cholesterol-modified Toll-like receptor 9 (TLR9) agonist oligonucleotide; CS—chitosan; DA-deoxycholic acid; DDS—drug delivery system; DGC-N-deoxycholic acid glycol chitosan; DSPE-PEG_2000_-1,2-distearoyl-sn-glycero-3-phosphoethanolamine-N-[amino(poly(ethylene glycol)-2000]; EE—encapsulation efficiency; FA—folic acid; Gba—galbanic acid; GNRs-gold nanorods; GXI—gastric cancer angiogenesis marker peptide; HA—hyaluronic acid; HSPC—hydrogenated phosphatidylcholine; PBAE—poly(β-amino ester); PCL—poly(ε-caprolactone); PEG—poly(ethylene glycol); PLA—poly(lactide); PLGA—poly(lactide-co-glycolide); PLGA-ATRA—poly(lactide-co-glycolide) all-trans-retinoic acid; PM—platelet membrane; Ptx—paclitaxel; RLT—low-density lipoprotein receptor (LDLR)-binding peptide; SPC—phosphatidylcholine; STTP—chitosan and sodium tripolyphosphate; Zol—zoledronate.

**Table 2:** Clinical trials of Dtx-loaded NPs based on https://clinicaltrials.gov/ (accessed on 10 February 2021).

Nr	Study Title	Cancer	DDS	Phase	Status
NCT01300533	A Study of BIND-014 Given to Patients with Advanced or Metastatic Cancer	Metastatic cancer, solid tumours	NPs	1	C
NCT02479178	A Study of BIND-014 in Patients with Urothelial Carcinoma, Cholangiocarcinoma, Cervical Cancer and Squamous Cell Carcinoma of the Head and Neck (iNSITE2)	Urothelial carcinoma cholangiocarcinoma, cervical cancer, squamous cell carcinoma of head and neck	NPs	2	T
NCT02283320	A Study of BIND-014 (Docetaxel Nanoparticles for Injectable Suspension) as Second-line Therapy for Patients with KRAS Positive or Squamous Cell Non-Small Cell Lung Cancer	KRAS-positive patients with non-small cell lung cancer, squamous cell non-small cell lung cancer	NPs	2	C
NCT01792479	A Phase 2 Study to Determine the Safety and Efficacy of BIND-014 (Docetaxel Nanoparticles for Injectable Suspension) as Second-line Therapy to Patients with Non-Small Cell Lung Cancer	Non-small cell lung cancer	NPs	2	C
NCT01812746	A Phase 2 Study to Determine the Safety and Efficacy of BIND-014 (Docetaxel Nanoparticles for Injectable Suspension), Administered to Patients with Metastatic Castration-Resistant Prostate Cancer	Castration-resistant prostate cancer, prostate cancer	NPs	2	C
NCT01151384	Liposome Encapsulated Docetaxel (LE-DT) in Patients with Solid Tumours (LE-DT)	Solid tumours	Liposomes	1	C
NCT01186731	Efficacy and Safety Study of LE-DT to Treat Locally Advanced or Metastatic Pancreatic Cancer	Pancreatic cancer	Liposomes	2	C
NCT01188408	Efficacy and Safety Study of LE-DT to Treat Metastatic Castrate Resistant Prostate Cancer	Prostate cancer	Liposomes	2	W
NCT01103791	A Trial to Determine the Maximum Tolerated Dose and Evaluate the Safety and Pharmacokinetics of Docetaxel-PNP, Polymeric Nanoparticle Formulation of Docetaxel, in Subjects with Advanced Solid Malignancies	Advanced solid malignancies	NPs	1	C
NCT03712423	PET Study With [89Zr]-Df-CriPec^®^ Docetaxel	Solid tumour	CCL-PMs	1	C
NCT03742713	Efficacy Study of CPC634 (CriPec^®^ Docetaxel) in Platinum Resistant Ovarian Cancer (CINOVA)	Cancer, ovarian cancer	CCL-PMs	2	C
NCT02442531	A Study of CriPec^®^ Docetaxel Given to Patients with Solid Tumours (NAPOLY)	Cancer, metastatic cancer, solid tumours	CCL-PMs	1	C

CCL-PMs—core-crosslinked polymeric micelles, C—completed, NPs—nanoparticles; T—terminated, W—withdrawn.

**Table 3:** The examples of Res–loaded delivery systems for anticancer therapy.

DDS	Material	Size [nm]	EE [%]	Preparation Method	Location	Status	Ref.
NPs	Au	30.75 ± 3.41	n/d	Reduction with chloroauric acid	Breast	In vitro—MCF-7 cells	[133]
NPs	PCL	132 ± 4 ^a^	98.4 ± 0.3 ^a^	Interfacial deposition	Skin	In vitro—B16F10 cells In vivo—mice	[134]
NPs	PLC, PLGA, PEG	150	83.30 ± 13.47	Nanoprecipitation	Prostate	In vitro—DU-145, PC-3 and LNCaP cells	[135]
NPs	PLGA	202.8 ± 2.64	89.32 ± 3.51	Solvent displacement	Prostate	In vitro—LNCaP cells	[136]
NPs	CB	n/d	n/d	N/d	Lung	In vitro—A549 cells	[137]
NPs	FA-HSA	102.1 ± 4.9	98.36	High pressure fluid nano-homogeneous emulsification	Liver	In vitro—HepG2 cells In vivo—mice	[138]
NPs	Pluronic F127 block copolymer, vitamin E-TPGS	179 ± 22	73 ± 0.9	Emulsification	Breast	In vitro—MCF-7, MDA-MB-231, MCF-10A cells	[139]
SLNPs	SA, saturated monoacid, triglyceride, Myrj52	168 ± 10.7	n/d	Emulsification and low-temperature solidification	Breast	In vitro—MDA-MB-231 cells	[140]
SLNPs	Apolipoprotein E, DSPE, palmitic acid	217.1 ± 5.8	98.9 ± 0.6	High shear homogenization	Brain	hCMEC/D3 cells	[141]

Au—gold; CB—carbon black; CS—chitosan; DDS—drug delivery system; DSPE—1,2-distearoyl-sn-glycero-3-phosphorylethanolamine; FA—folic acid; HSA—human serum albumin; Myrj52-polyoxyethylene (40) stearate; PCL—poly(ε-caprolactone); PLGA—poly(lactide-co-glycolide); SA—stearic acid. ^a^ nanoparticles containing 10 mg of Res.

**Table 4:** Examples of Res and Dtx-loaded delivery systems for anticancer therapy.

DDS	Material	Preparation Method	Location	Status	Ref.
micelles	mPEG-PDLA	Thin film hydration-ultrasound method	Breast	In vitro—MCF-7 cells In vivo—rats	[71]
PBM NPs	FA-PCL-PEG	Planetary ball milling	Prostate	In vitro—PC3 and PC3-R cells	[151]
LPNPs	Lipid-polymer	Nanoprecipitation method	Lung	In vitro—HCC827, NCIH2135 and HUVEC cells In vivo—mice	[156]

FA—folic acid; PCL—poly(ε-caprolactone); mPEG-PDLA-methoxyl poly(ethylene glycol)-poly(D,L-lactide); PEG—poly(ethylene glycol); PBM NPs—planetary ball milled nanoparticles; LPNPs—lipid polymer nanoparticles.

## Data Availability

Not applicable.

## Abbreviations

4T1 cells	murine mammary carcinoma cells
γ-PGA	poly(γ-glutamic acid)
A549 cells	human epithelial carcinoma cell line
ACUPA	((S)-2-(3-((S)-5-amino-1-carboxypentyl) ureido) pentanedioic acid
AMPK	AMP-activated protein kinase
AP-1	activator protein-1
ATRA	all-trans-retinoic acid
Au	gold
B16F10 cells	melanoma lung metastasis cell line
B6 cells	melanoma lung metastasis cell line
A375 cells	melanoma lung metastasis cell line
BSA	bovine serum albumin
BT-474 cells	human breast cancer cell line
CA	caffeic acid
Caco-2 cells	colon carcinoma cell line
Cap	calcium phosphate
CB	carbon black
CHOL	cholesterol
cho-CpG	cholesterol-modified Toll-like receptor 9 (TLR9) agonist oligonucleotide
COX	cyclooxygenase enzymes
COX-2	cyclooxygenase-2 enzymes
CS	chitosan
CRPC cells	castration-resistant prostate cancer cell line
CT26 cells	murine colorectal carcinoma cell line
Cur	curcumin
DA	deoxycholic acid
DDS	drug delivery system
DGC	N-deoxycholic acid glycol chitosan
Dha	dihydroartemisin
DLT	dose limiting toxicity
DSPE	1,2-distearoyl-sn-glycero-3-phosphorylethanolamine
DSPE-PEG2000	1,2-distearoyl-sn-glycero-3-phosphoethanolamine-N-[amino(poly(ethylene glycol)-2000]
Dtx	docetaxel
Dtx-CCL-PMs	docetaxel-entrapped core-cross-linked polymeric micelles
DU-145 cells	docetaxel-resistant prostate carcinoma cell line
EGFR	epidermal growth factor receptor
eSM	egg sphingomyelin
FA	folic acid
FDA	Food and Drug Administration
FRα	folic acid α receptor
fWGA	fluorescein-labelled wheat germ agglutinin
G-CSF	granulocyte colony-stimulating factors
Gba	galbanic acid
GNRs	gold nanorods
GX1	gastric cancer angiogenesis marker peptide
H520 cell line	human lung squamous carcinoma cell lines
HA	hyaluronic acid
HCC827 cells	non-small cell lung cancer cell line
hCMEC/D3 cells	brain microvascular endothelial cell line
HepG2 cells	human hepatocellular carcinoma cell line
HIF-1α	hypoxia-induced factor
HSA	human serum albumin
HSPC	hydrogenated phosphatidylcholine
HSR	hypersensitivity reaction
HT-29 cells	human colorectal adenocarcinoma cell line
HUVEC cells	human umbilical vein endothelial cell line
IL-17	interleukin-17
LNCaP	prostate cancer cells
LPNPs	lipid-polymer nanoparticles
MAPK	p38-Mitogen activated protein kinase
MC-38 cells	murine colon adenocarcinoma cell line
MCF-7	human breast carcinoma cell line
MCF-10A cells	non-malignant breast epithelial cell line
MDA-MB-231 cells	human breast adenocarcinoma cell line
MDA-MB-453 cells	human breast adenocarcinoma cell line
MDR	multidrug resistance
mEHT	modulated electro-hyperthermia
MMPs	metalloproteinases
mPEG-PDLA	methoxyl poly(ethylene glycol)-poly(D,L-lactide)
MTD	maximum tolerated dose
Myrj52	polyoxyethylene (40) stearate
NCIH2135 cells	non-small cell lung cancer cell line
NF-κB	nuclear transcription factor-κB
NIR	near-infrared radiation
NPs	nanoparticles
NSCLC	non-small cell lung cancer
P-gp	P-glycoprotein
PAMAM	poly(amidoamine)
PAMAMOS	PAMAM-organosilicon
PBAE	poly (β-amino ester)
PBM	planetary ball milled
PBMC	peripheral blood mononuclear cells
PBS	phosphate-buffered saline
PC-3 cells	human caucasian prostate adenocarcinoma cell line
PC-3–R cells	resistance human caucasian prostate adenocarcinoma cell line
PCL	poly(ε-caprolactone)
PEG	poly(ethylene glycol)
PDLA	poly(D,L-lactide)
PLA	poly(lactide)
PLGA	poly(lactide-co-glycolide)
PLGA-ATRA	poly(lactide-co-glycolide) all-trans-retinoic acid
PM	platelet membrane
PNT2 cells	normal human prostate cell line
PPI	poly(propylene imine)
PTT	photo-thermal therapy
Ptx	paclitaxel
Res	resveratrol
Res-AuNPs	Res-loaded gold nanoparticles
RLT	low-density lipoprotein receptor (LDLR)-binding peptide
ROS	reactive oxygen species
SA	stearic acid
SHBG	sex steroid hormone-binding globulin
SIRT1	sirtuins signalling pathway
SKBR-3 cells	human breast adenocarcinoma cell line
SPC	phosphatidylcholine
SLNPs	solid lipid nanoparticles
STTP	chitosan and sodium tripolyphosphate
TF	transferrin
TNF-α	tumour necrosis factor
VEGF	vascular endothelial growth factor
Zol	zoledronate
